# Application of Genome-Assisted Prediction (GAP) for Apple Fruit Weight

**DOI:** 10.3390/plants15010065

**Published:** 2025-12-25

**Authors:** Meng Yu, Yingying Yang, Bin Liang, Yujia Peng, Li Liu, Yazhou Yang

**Affiliations:** State Key Laboratory for Crop Stress Biology for Arid Areas, College of Horticulture, Northwest A&F University, Yangling 712100, China; 2023055225@nwafu.edu.cn (M.Y.); yyy17686260569@163.com (Y.Y.); 2024055364@nwafu.edu.cn (B.L.); 2024055361@nwafu.edu.cn (Y.P.); liulily@nwafu.edu.cn (L.L.)

**Keywords:** apple, fruit weight, SNP, marker combination, GAP accuracy

## Abstract

Apple fruit weight is a critical quantitative trait controlled by multiple genes, with numerous quantitative trait loci (QTLs) having been identified. This study evaluated the potential of genome-assisted prediction (GAP) by genotyping 573 hybrids from six populations with 70 previously identified quantitative trait locus (QTL)-based markers. Genetic diversity and marker-trait association (MTA) analyses identified two optimized marker combinations. Results showed that using the screened marker combination with moderate PIC (Polymorphism Information Content, PIC) and HWE-conforming (Hardy–Weinberg Equilibrium, HWE) significantly increased accuracy compared to the full marker set in specific populations, such as ‘YM1’ × ‘Honeycrisp’ (Y × H; r increased from 0.115 to 0.204; *p* < 0.05) and ‘Fuping Qiuzi’ × ‘Ruixue’ (Q × RX; r increased from 0.482 to 0.576; *p* < 0.001). MTA-based (marker-trait association, MTA) marker combinations achieved the highest accuracy in most populations, particularly in Q × RX (r = 0.5766, *p* < 0.001) and Y × H (r = 0.2475, *p* < 0.05). In contrast, the full marker set showed population dependence (r ranging from −0.088 to 0.482). These results indicate that the 70 markers were not generally effective across diverse apple hybrids. We propose a procedure for screening effective markers (PIC, HWE, MTA), providing a practical framework and theoretical foundation for implementing GAP in apple breeding.

## 1. Introduction

Apple (*Malus* × *domestica* Borkh.) is a globally significant temperate fruit tree, with world production exceeding 90 million tons in 2023 [1]. Breeding superior apple varieties with desirable traits is a key focus for researchers and producers worldwide [2]. Among critical agronomic targets, fruit size (weight) directly impacts yield and appearance quality [3,4]. As a key visual quality parameter, fruit size significantly influences consumer purchasing decisions [5] and post-harvest processing efficiency, where sorting systems classify apples by size for pricing stratification [6]. Generally, larger fruits command premium prices in fresh markets, whereas smaller sizes yield diminished economic returns [6].

Apple fruit weight is under complex genetic control, being a quantitative trait governed by multiple genes [7,8]. Previous studies have mapped numerous SNPs, QTLs, and MetaQTLs for fruit weight across all 17 chromosomes of apple [9,10,11,12,13,14]. Liebhard et al. [15] identified genomic regions associated with genetic control of fruit weight in the ‘Fiesta’ × ‘Discovery’ hybrid population on chr1, chr3, chr6, chr8, chr10, chr12, chr15, chr16. Kenis K et al. [16] identified QTLs for fruit weight on Linkage Groups (LGs) in a genetic linkage map of the F_1_ population of ‘Telamon’ × ‘Braeburn’, primarily located on LGs 6, 9, 10, 17. Devoghalaere et al. [14] identified six fruit weight QTLs on LGs 5, 8, 8, 11, 15, 16, and 17, using genetic maps in two populations: ‘Royal Gala’ × ‘Braeburn’ (RG × BB) and ‘Starkrimson’ × ‘Granny Smith’ (STK × GS), with three of these QTLs involved in an epistatic effect. Minamikawa et al. [11], using 21 breeding lines of apple, identified five significant loci for fruit weight on chr05, chr11, chr13, and ch15 bya genome-wide association study (GWAS) based on SNP genotypes. Costa et al. [13] reported two MetaQTLs for fruit weight, namely MetaQTL4.2 and MetaQTL7.2, using four hybrid populations: ‘Fuji’ × ‘Delearly’, ‘Fuji’ × ‘Cripps Pink’, ‘Golden Delicious’ × ‘Scarlet’, and ‘Golden Delicious’ × ‘Braeburn’. Shen et al. [9] mapped 90 fruit weight QTLs across 10 chromosomes using the three hybrid populations ‘Jonathan’ × ‘Golden Delicious’ (J × G), ‘Zisai Pearl’ × ‘Red Fuji’ (Z × F), and ‘Zisai Pearl’ × ‘Golden Delicious’ (Z × G), with chr14 being a novel discovery. However, the effects of these loci are often small, susceptible to environmental influences and genetic background, and subject to complex epistatic effects [17,18].

Beyond QTL mapping, studies on specific genes have elucidated the molecular mechanisms underlying fruit size variation. Genetic variation in cell division-related genes is closely associated with apple fruit size. Both *SNP379 G/T* and the functional variations in the promoter of *MdSAUR36* contribute to the small-fruit trait [19]. Post-transcriptional accumulation of *miRNA172p* negatively regulates fruit growth by suppressing cell division during early development and cell expansion throughout developmental stages, ultimately reducing fruit size [20]. In a study of auxin’s role in apple fruit size, a fruit weight QTL co-localized with the auxin response factor gene *ARF106*. Given its high expression during both cell division and expansion phases, this QTL was suggested to encompass genes regulating the cell cycle, thereby promoting increased fruit size [14]. However, unlike the tomato with its major gene *FW2.2*, no single major gene exerting a similarly significant effect on fruit size has been identified in apples to date.

Conventional apple breeding relies heavily on phenotypic selection, a straightforward yet subjective approach influenced by genetic, environmental, and cultivation factors. Furthermore, due to the prolonged juvenile phase in apple, breeding cycles are protracted, taking approximately 20 years via conventional breeding methods to develop a new variety [21], with relatively high associated costs. With the completion of apple genome sequencing [22] and advances in molecular marker technology, research in apple molecular biology has advanced rapidly. Marker-assisted selection (MAS) has been increasingly applied to apple breeding [23,24]. MAS is applicable to qualitative traits controlled by single or major genes. However, as apple has relatively few qualitative traits and most breeding targets are quantitative traits influenced by non-genetic factors (e.g., environment), the application scope of MAS is limited. Recent developments in sequencing technology have expanded the number of available markers and reduced the cost of high-density genome-wide single nucleotide polymorphism (SNP) genotyping [25]. Genomic selection (GS) selects superior genotypes based on genome estimated breeding values (GEBV) derived from genome-wide marker information [26] and is more effective than MAS, particularly for fruit traits controlled by many minor-effect genes [27,28]. Although genotyping costs have decreased substantially over the past decade, genotyping thousands of individuals remains an economic barrier to widespread application. Shen et al. [9] used the bulked segregant analysis tool for outcrossing species (BSATOS) to identify 90 significant QTLs for apple fruit weight, along with predicted candidate genes. They developed 71 QTL-based markers and genotyped 1396 individuals in a training population. Using marker genotype effect estimates from this population, they trained a GAP model for fruit weight, achieving a prediction accuracy of 0.7658 validated by five-fold cross-validation. This accuracy was relatively higher than most reported pure GS accuracies (0.18–0.70) obtained using a high-density SNP array. GAP can achieve comparable or better accuracy than GS, but at a lower cost. GAP, integrating aspects of MAS and GS, can effectively assist breeding efforts in apples and other species. For instance, GAP accuracy for the retainability of flesh firmness and crispness was superior to that of both the activator-depleted substrate model and pure GS using ridge regression BLUP [29]. Furthermore, the average prediction accuracy of GAP for fruit weight was higher than that of rr-BLUP [9]. Although the GAP model based on 71 QTL-based markers has been developed, the general applicability of QTL-based markers and the GAP model across diverse breeding populations remains unclear.

Given the importance of fruit size in apple breeding, it is essential to validate the effectiveness of these genetic markers and the accuracy of GAP across diverse breeding materials. In this study, six hybrid populations were used as validation populations. Using SNP genotyping results from 70 previously developed QTL-based markers related to apple fruit weight by Shen et al. [9], we evaluated marker effectiveness through genetic diversity analysis and marker-trait association analysis. Furthermore, Pearson’s correlation coefficient between the Genomic Prediction Value (GPV) and Observed Phenotypic Value (OPV) was calculated across populations to assess the accuracy of GAP. This study aims to verify the applicability of the QTL-based markers and GAP models previously developed by Shen et al. [9], evaluate the application potential of GAP, provide practical suggestions for its implementation in apple breeding and offer a theoretical reference for the genetic improvement of complex traits in apple.

## 2. Results

### 2.1. Evaluation of Fruit Weight Phenotypic Traits

**Figure 1:**
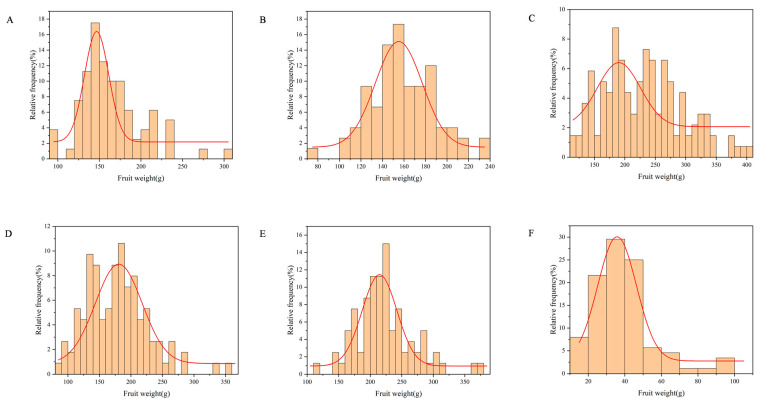
Relative frequency distribution of fruit weight in hybrids. (**A**) ‘Ralls Janet’ × ‘Ruiyang’. (**B**) ‘Ralls Janet’ × ‘YM1’. (**C**) ‘YM1’ × ‘Honeycrisp’. (**D**) ‘Ralls Janet’ × ‘Honeycrisp’. (**E**) ‘Ruixue’ × ‘Alps Otome’. (**F**) ‘Fuping Qiuzi’ × ‘Ruixue’.

According to the single-sample Kolmogorov–Smirnov test (Figure 1), the fruit weight distribution in ‘Fuping Qiuzi’ × ‘Ruixue’ (Q × RX) deviated from normality (*p* < 0.05), while the distributions of the other five hybrids followed a normal distribution (*p* > 0.05). As illustrated in Table 1, fruit weight in the F1 generation exhibited continuous variation, consistent with the genetic characteristics of quantitative traits. The widest range of variation was observed in ‘YM1’ × ‘Honeycrisp’ (Y × H), from 116.14 to 405.55 g, indicating the highest degree of dispersion. The maximum coefficient of variation (CV) was in Q × RX (44.11%), reflecting substantial differences in parental genetic backgrounds. Q × RX also showed the lowest heterosis rate (H) of −73.69% and a high ratio of individuals with fruit weight lower than low parent (RL) of 31.82%, indicating significant negative heterosis. Positive heterosis was observed only in ‘Ruixue’ × ‘Alps Otome’ (RX × A), with an H of +44.75%, while heterosis was negative in the remaining crosses. RX × A showed the highest genetic transmitting ability (Ta) of 144.75%, while Q × RX had the lowest at 26.31%.

### 2.2. SNP Genotyping Results and Genetic Diversity Analysis

**Figure 2:**
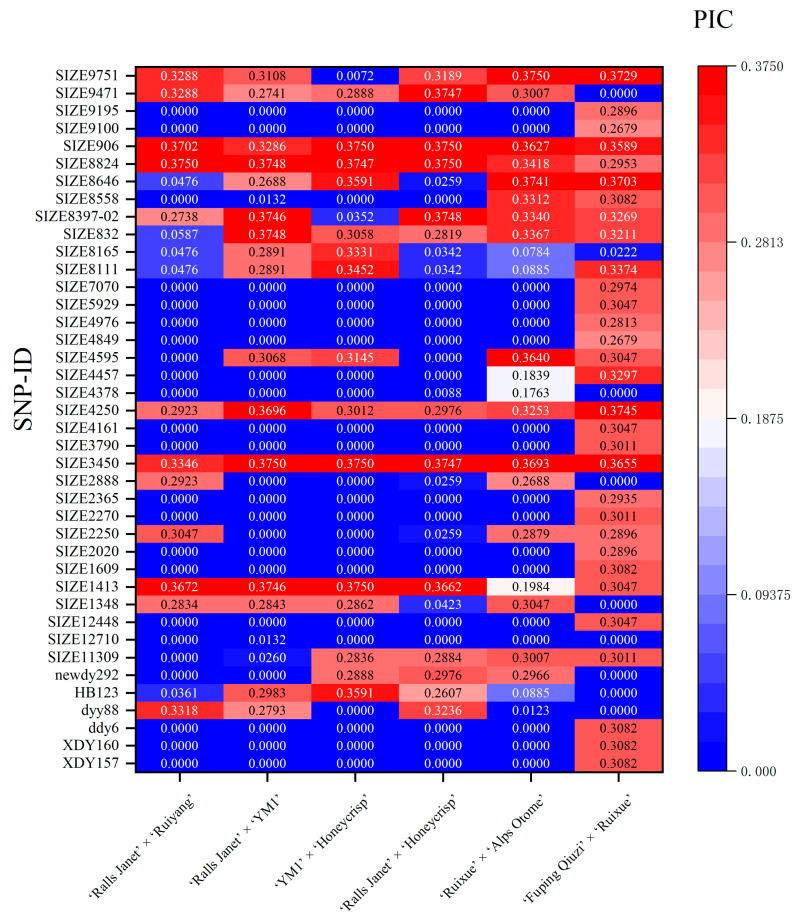
Polymorphic information content (PIC) of SNP loci associated with apple fruit weight in hybrid populations, ‘Ralls Janet’ × ‘Ruiyang’, ‘Ralls Janet’ × ‘YM’, ‘YM1’ × ‘Honeycrisp’, ‘Ralls Janet’ × ‘Honeycrisp’, ‘Ruixue’ × ‘Alps Otome’, ‘Fuping Qiuzi’ × ‘Ruixue’.

**Figure 3:**
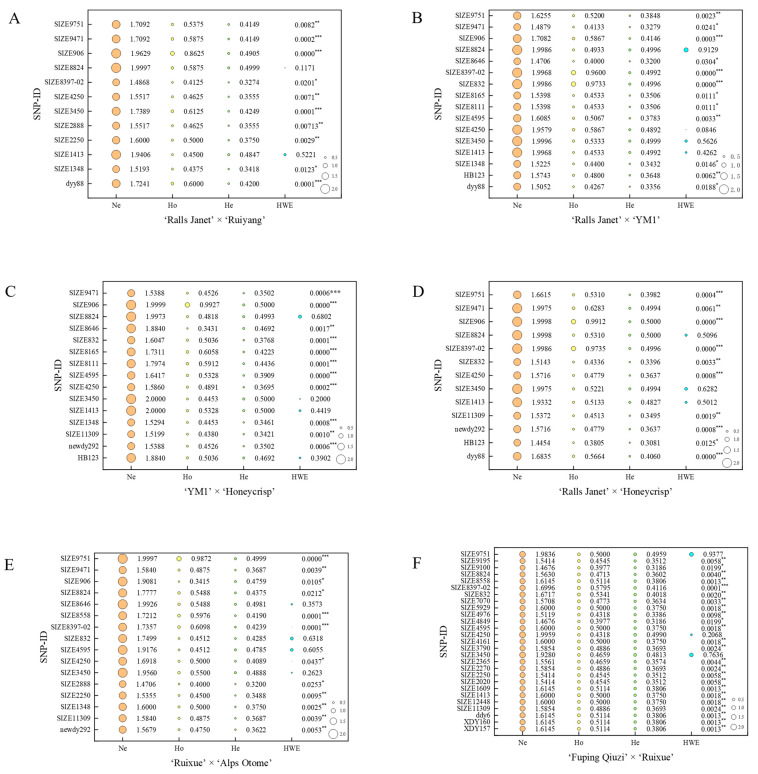
SNP loci of each hybrid and their genetic diversity analysis. (**A**) ‘Ralls Janet’ × ‘Ruiyang’. (**B**) ‘Ralls Janet’ × ‘YM1’. (**C**) ‘YM1’ × ‘Honeycrisp’. (**D**) ‘Ralls Janet’ × ‘Honeycrisp’. (**E**) ‘Ruixue’ × ‘Alps Otome’. (**F**) ‘Fuping Qiuzi’ × ‘Ruixue’. Ne: the number of effective alleles; Ho: observed heterozygosity; He: expected heterozygosity; HWE: Hardy–Weinberg equilibrium *p*-value (* indicates *p* < 0.05, ** indicates *p* < 0.01, *** *p* < 0.001).

Of the 70 markers examined, 40 loci were polymorphic. The polymorphism information content (PIC) at a given locus was population-dependent; a marker segregating in one population might not do so in another. The results (Figure 2 and Figure 3; Table S3) showed: In ‘Ralls Janet’ × ‘Ruiyang’ (RJ × RY) (Figure 3A), there were 28 low-polymorphism SNP loci (PIC < 0.25) and 12 moderately polymorphic SNP loci (0.25 ≤ PIC < 0.5); among the moderately polymorphic loci, 8 conformed to Hardy–Weinberg equilibrium (HWE, *p* > 0.001), while 4 deviated significantly (*p* < 0.001). In ‘Ralls Janet’ × ‘YM1’ (RJ × Y) (Figure 3B), there were 24 low-polymorphism SNP loci and 16 moderately polymorphic loci; among the moderately polymorphic loci, 13 loci conformed to HWE (*p* > 0.001), while 3 deviated significantly (*p* < 0.001). In Y × H (Figure 3C), there were 25 low-polymorphism SNP loci and 15 moderately polymorphic SNP loci; among the moderately polymorphic loci, 6 loci conformed to HWE (*p* > 0.001), while 9 deviated significantly (*p* < 0.001). In ‘Ralls Janet’ × ‘Honeycrisp’ (RJ × H) (Figure 3D), there were 27 low-polymorphism SNP loci and 13 moderately polymorphic SNP loci; among the moderately polymorphic loci, 7 loci conformed to HWE (*p* > 0.001), while 6 deviated significantly (*p* < 0.001). In RX × A (Figure 3E), there were 24 low-polymorphic SNP loci and 16 moderately polymorphic loci; among the latter, 13 conformed to HWE (*p* > 0.001) and 3 deviated significantly (*p* < 0.001). In Q × RX (Figure 3F), there were 13 low-polymorphic SNP loci and 27 moderately polymorphic SNP loci; among the latter, 26 loci conformed to HWE (*p* > 0.001), while 1 deviated significantly (*p* < 0.001).

PIC values ranged from 0 to 0.5 across populations. As no high-polymorphism locus were found, using moderate polymorphism as a screening criterion helps eliminate loci with low information content. Deviation from HWE may indicate typing errors, so loci with severe deviation (*p* < 0.001) were eliminated. Ultimately, marker combinations with PIC > 0.25 and HWE-conforming were selected as listed in Table S5 (Table S5 lists, for each cross, (i) the full 70-marker set, (ii) the PIC > 0.25 & HWE-conforming subset, and (iii) the MTA-based subset, together with the exact markers comprising each combination).

### 2.3. Marker-Trait Association Analysis

**Figure 4:**
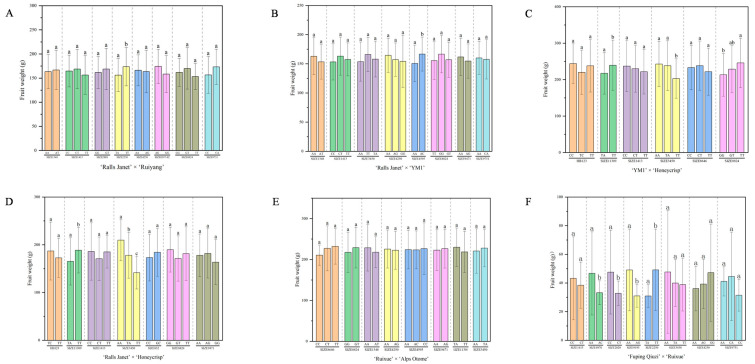
Significance of marker-trait association (MTA) for fruit weight in hybrid populations. (**A**) ‘Ralls Janet’ × ‘Ruiyang’. (**B**) ‘Ralls Janet’ × ‘YM1’. (**C**) ‘YM1’ × ‘Honeycrisp’. (**D**) ‘Ralls Janet’ × ‘Honeycrisp’. (**E**) ‘Ruixue’ × ‘Alps Otome’. (**F**) ‘Fuping Qiuzi’ × ‘Ruixue’. Different lowercase letters (a, b, c) above the bars indicate statistically significant differences among genotypic groups at *p* < 0.05 based on post hoc tests. Significance marks denote FDR q < 0.05 (see Table S7). Genomic positions of MTA-based SNPs are in Table S6.

Significance analysis was conducted on phenotypic values associated with genotypes at loci exhibiting moderate polymorphism and conforming to HWE using IBM SPSS Statistics 20.0 (Table S4). To account for the multiple comparisons performed across all marker loci, the False Discovery Rate (FDR) was controlled using the Benjamini–Hochberg procedure. Marker trait association (MTA) significance was declared at BH-FDR adjusted q < 0.05; corresponding raw *p*-values and q-values are summarized in Table S7. The results showed that in RJ × RY (Figure 4A), there was a significant MTA (*p* < 0.05, q < 0.05) for locus SIZE2250, while there was no significant difference (*p* > 0.05) for SIZE1348 and the other six loci. In RJ × Y (Figure 4B), there was a significant MTA (*p* < 0.05, q < 0.05) for SIZE4595; taking SIZE1348 as an example, no significant associations (*p* > 0.05) were detected for SIZE1348 or the other 11 loci. In Y × H (Figure 4C), significant MTAs (*p* < 0.05, q < 0.05) were detected for SIZE11309, SIZE3450, and SIZE8824; no significant associations (*p* > 0.05) were found for HB123 or the other two loci. In RJ ×H (Figure 4D), significant MTA (*p* < 0.05) were detected for SIZE11309 and SIZE3450; no significant associations (*p* > 0.05) were found at five loci such as HB123. In RX × A (Figure 4E), none of the 13 loci tested showed significant MTAs. In Q × RX (Figure 4F), significant MTAs (*p* < 0.05, q < 0.05) were detected for 10 loci, ddy6, SIZE12448, SIZE4976, SIZE2020, SIZE9195, SIZE2250, SIZE2270, SIZE2365, SIZE3790, SIZE4161; no significant associations were found for the remaining 16 loci (e.g., SIZE1413, *p* > 0.05). Ultimately, the MTA loci constituted MTA-based marker combinations (Table S5). Genomic physical positions for all MTA-based SNPs are provided in Table S6 to facilitate downstream candidate-gene discovery.

### 2.4. Accuracy of GAP

The correlation coefficient between GPV and OPV was calculated to evaluate prediction accuracy. Marker combinations used for GAP analysis in each hybrid cross had been listed in Table S5. For the fruit weight trait (Table 2), using all 70 markers, the correlations between GPV and OPV in RJ × RY, RJ × Y and RX × A were all close to zero, indicating low prediction accuracy. Using the marker combination that screened for PIC > 0.25 and HWE-conforming resulted in correlation coefficients still near zero. However, using the significantly associated marker combination yielded higher absolute correlation coefficients, around 0.2, except for RX × A (due to the absence of MTA markers). Using all 70 markers, GAP prediction in Q × RX showed an extremely significant positive correlation (*p* < 0.001), r = 0.4819; using the 8 screened PIC > 0.25 and HWE-conforming markers, the correlation increased to r = 0.5755 (*p* < 0.001); using the 10 significantly associated markers yielded a further increase to r = 0.5766 (*p* < 0.001), representing the highest prediction accuracy among the populations. In Y × H, using all 70 markers gave r = 0.1152; using the 6 screened PIC > 0.25 and HWE-conforming markers increased the correlation to r = 0.2035 (*p* < 0.05); using the 3 significantly associated markers increased it to r = 0.2475 (*p* < 0.05). In RJ × H, using all 70 markers gave r = 0.1238; using the 7 screened PIC > 0.25 and HWE-conforming markers decreased the correlation to r = 0.1010; using the 2 significantly associated markers further increased it to r = 0.1943. The results indicate that the screened marker combinations could improve prediction accuracy in specific populations.

## 3. Discussion

### 3.1. Genetic Analysis of Marker Performance Across Populations

Genetic analysis of the genotyping results revealed that the 70 QTL-based markers exhibited differential segregation patterns across the six hybrid populations. For instance, locus SIZE2250 had a PIC value of 0 indicating no segregation in the RJ × Y and Y × H, while it segregated in the other four hybrids. This population-specific segregation is consistent with the findings of Shen et al. [9] and underscores that the informativeness of a marker is highly dependent on the genetic background of the population under study.

Among the populations, Q × RX, which involved the wild species *Malus prunifolia* (Willd.) Borkh. (MPB) and a commercial cultivar, exhibited the highest number of segregating loci (27), the highest phenotypic coefficient of variation (44.11%), and the greatest number of markers showing a significant marker-trait association (10). In contrast, populations derived solely from commercial cultivars, such as RX × A, showed fewer segregating loci and no significant MTAs. This pattern aligns with the expected parental genetic distance and supports the view that intensive selection and clonal propagation in cultivated apples (*Malus × domestica*) have led to a narrower genetic base compared to the broader gene pool that includes wild relatives [8,30,31]. The higher genetic diversity and marker utility observed in Q × RX highlight the value of introducing wild germplasm into breeding programs to uncover functional genetic variation.

### 3.2. Application and Performance of QTL-Based Markers and GAP Model

PIC values reflect the informativeness of a marker locus; a low PIC indicates a limited ability to discriminate among genotypes within a population. HWE describes a model with stable genotype frequencies in an ideal population. Loci that deviate from HWE may suggest inbreeding, selective stress or strong population structure. Strong population structure or inbred individuals may violate the assumption of random mating [32]. Loci deviating from HWE are often flagged in data quality control, potentially influenced by selection pressure or gene flow, and may reflect hidden technical or biological issues [33,34]. Low-polymorphism and significantly HWE-deviated SNP loci should be used cautiously in prediction models. Given the observed population specificity of marker segregation, we implemented a pre-screening strategy to identify effective marker subsets for GAP. Marker combinations were screened based on PIC > 0.25 and HWE-conforming to ensure they contained relatively rich polymorphic information and excluded loci violating the random mating hypothesis. Screening based on MTA ensured the effectiveness and reliability of the markers. The goal was to retain markers with sufficient informativeness, reliable population genetics properties, and a direct statistical link to the target trait within the specific validation population.

This screening proved valuable. For hybrid populations like RJ × RY, the correlation using all 70 markers was close to 0, indicating low prediction accuracy. In most hybrid populations, the correlation using the full set of 70 markers was the lowest among the three marker combinations, suggesting that the inclusion of redundant or uninformative markers may impair prediction accuracy. Consistent with this, Jurado-Ruiz et al. [35] demonstrated that while a substantial number of SNPs is essential, the inclusion of additional, unrelated SNPs degrades performance. The screened marker combinations consistently improved accuracy. For example, when using the marker combination based on PIC > 0.25 and HWE-conforming, the correlation increased significantly compared to the full marker set in Y × H and Q × RX. Furthermore, the MTA-based subset yielded the highest accuracy among all three marker combinations in five out of six populations. This demonstrates that a targeted subset of well-chosen, population-specific QTL markers can achieve higher predictive accuracy than a fixed, generic panel.

Li et al. [36] identified nine and seven major-effect markers for the small- and large-fruit phenotypes, respectively, derived from previously identified 90 QTLs for apple fruit weight via bulked segregant analysis-seq. At locus Chr02_23 876 017, the AA, CA, CC genotypes exhibited statistically significant differences in fruit weight, which followed the descending order of CC > CA > AA. For the large-fruit phenotype, they divided the original large-fruit subset (L) of the training population into three redefined groups: medium (M), large-medium (LM), and large (L) fruit phenotypes. Individuals carrying G allele at locus Chr13_13 933 420 were classified into the medium-fruit group, with an average fruit weight of 81.64 g (SD = 22.0 g). This indicates that the G allele at Chr13_13 933 420 is significantly associated with reduced fruit size [36]. Chr02_23 876 017 and Chr13_13 933 420 were identified as major-effect markers for small- and large-fruit traits, corresponding to SIZE12448 and SIZE2365 (Table S6), respectively. Both SIZE12448 and SIZE2365 were MTA markers in Q × RX in this study, but their genotypes were monomorphic in other populations, explaining their lack of significance there and emphasizing the critical role of genetic background. The genotype–phenotype association we observed (Figure 4F) aligns with the results proposed by Li et al. [36], supporting their major effects. This indicates that our method of analyzing the effectiveness of the markers is feasible. Further validation is needed to assess the major-effect status of the remaining MTA-based loci.

Moreover, our results confirm that the 70 SNP markers evaluated exhibit population-specific applicability and are not generally effective across diverse apple hybrid populations. Furthermore, we speculate that the SNP markers mapped to QTLs identified in training populations ‘Zisai Pearl’ × ‘Red Fuji’ (Z × F) and ‘Zisai Pearl’ × ‘Golden Delicious’ (Z × G) may have broader applicability than those from ‘Jonathan’ × ‘Golden Delicious’ (J × G) in our validation populations, as loci showing MTA in multiple populations were all mapped to the QTLs identified in Z × F and J × G (Table S6). The highest prediction accuracy was achieved in Q × RX (r = 0.5766, using 10 MTA markers), demonstrating the potential of GAP with a small, optimized marker set. These findings align with previous suggestions that incorporating known QTL information can enhance prediction in genomic-assisted models [9,37,38,39,40]. Consequently, the core contribution of this work lies in proposing and validating a general pre-screening framework that integrates genetic diversity (PIC/HWE) and MTA. This strategy enables the efficient identification of high-value marker subsets tailored to target populations from larger marker panels, thereby optimizing GAP prediction accuracy and providing a practical framework for apple breeding.

### 3.3. Factors Influencing the Accuracy of GAP

The prediction accuracy of GAP is influenced by the genetic structure and the relatedness between the training and validation populations [9,41,42]. Our study observed a wide range of prediction accuracies (−0.088 to 0.4819) using the full marker set, highlighting this dependency. Notably, the exceptionally high accuracy in the Q × RX population is likely attributable to the close genetic relationship between ‘Fuping Qiuzi’ (MPB) and ‘Zisai Pearl’ (*Malus asiatica* Nakai, MAN), the latter being a key parent in the original training population used for QTL discovery [9]. In Q × RX, MTA loci were all located within QTL intervals previously identified in populations where ‘Zisai Pearl’ was a parent, such as ddy6, SIZE12448, SIZE3790 in ZF-Z02.2, SIZE2020 and SIZE9195 in ZF-Z13.2, ZG-Z13.3, ZG-Z13.2. Gao et al. [43] concluded from the results of cluster analysis and principal component analysis that *M. asiatica* and *Malus prunifolia* belong to the same group. Furthermore, research on the phylogenetic relationships by Gao et al. [44] supports a close relationship between MAN and MPB, consistent with our interpretation. Further research with larger and more diverse validation populations is warranted to elucidate the impact of training-validation population relatedness on GAP accuracy.

Furthermore, the non-normal distribution of fruit weight in Q × RX (Figure 1F) may indicate particularities in its genetic architecture, such as the presence of loci with large effects. The identification of multiple significant MTA markers in this population, including the aforementioned major-effect candidates, provides circumstantial support for this hypothesis, though functional validation is required.

Although this study validated the effectiveness of the pre-screening strategy across multiple populations, future research could further deepen this work in the following directions: verifying the robustness of this strategy in larger populations with more complex genetic relatedness, identifying superior GAP solutions that can disentangle the influence of genetic background on prediction accuracy, and performing colocalization analysis between these population-specific effective markers and key candidate genes for fruit weight to elucidate the molecular mechanisms underlying fruit size variation in apple.

## 4. Materials and Methods

### 4.1. Plant Material and Orchard Locations

In this experiment, six hybrid populations were used as segregating populations, ‘Ralls Janet’ × ‘Ruiyang’ (RJ × RY), ‘Ralls Janet’ × ‘YM1’ (RJ × Y), ‘YM1’ × ‘Honeycrisp’ (Y × H), ‘Ralls Janet’ × ‘Honeycrisp’ (RJ × H), ‘Ruixue’ × ‘Alps Otome’ (RX × A) and ‘Fuping Qiuzi’ × ‘Ruixue’ (Q × RX). Among the parents, ‘Fuping Qiuzi’ is a wild species belonging to *Malus prunifolia* (Willd.) Borkh., while the other six parents are commercial cultivars belonging to *Malus × domestica* Borkh. ‘Fuping Qiuzi’ was used as a parent to introduce novel genetic variation into the breeding populations. All materials were grafted on M26 rootstock at a spacing of 0.5 × 3 m in autumn 2019 for the evaluation of apple quality traits.

The six hybrid populations were planted in the hybrid reselection orchard at the Northwest A&F University Apple Experimental Station in Baishui County, Weinan City, Shaanxi Province. All plant materials were subjected to standard orchard cultivation management, including flower and fruit thinning, as well as pest control. Phenotypic surveys were conducted during the 2022–2023 period. The hybrid crosses and their respective population sizes are presented in Table 3.

### 4.2. Phenotypic Measurement of Fruit Weight Trait

During the fruit ripening period, fruit maturity was determined based on ground color change (to yellow or white) and flavor assessment. Three to five mature fruits of uniform maturity were selected from different positions on the outer canopy [45]. Fruit weight was measured using an electronic balance with a precision of 0.01 g, and the average weight per tree was subsequently calculated. The phenotype data are presented in Table S1.

### 4.3. DNA Extraction and SNP Genotyping

Young, healthy leaves were collected from 573 individuals across the hybrid populations using a leaf hole punch. Immediately after collection, samples were sealed in airtight containers to preserve DNA integrity before being sent to Molbreeding (Shijiazhuang, Hebei, China) for high-throughput DNA extraction and genotyping.

Genomic DNA was extracted using a standardized cetyltrimethylammonium bromide (CTAB) method. SNP genotyping was performed on the Genotyping by Target Sequencing (GBTS) platform, a liquid-phase chip technology developed by Molbreeding. The specific panel employed was the MP0048_Mp_v5.0, which is derived from a patent that was subsequently developed into commercially available chips by Zhang et al. [46].

SNP genotyping was performed using the company’s proprietary bioinformatics pipeline, which included quality control, read alignment to the reference genome, and variant calling. The complete SNP genotyping results are provided in Table S2.

### 4.4. Genetic Analysis

Fruit weight data for each hybrid cross was compiled using Microsoft Excel 2019 to calculate relevant values. Normality testing and significance analysis of differences were conducted using IBM SPSS Statistics 20.0. Graphs were generated using OriginPro 2021. The following formulas were used for relevant calculations:

Coefficient of variation (CV): $CV(\%)=\left(\frac{S}{\bar{X}}\right)\times 100$; Genetic transmitting ability (Ta): $Ta(\%)=\left(\frac{\bar{X}}{MP}\right)\times 100$; Heterosis rate (H): $H(\%)=\left(\frac{\bar{X}-MP}{MP}\right)\times 100$; Ratio of higher than the high parent (RH): $RH(\%)=\left(\frac{n_{hp}}{n}\right)\times 100$; Ratio of lower than low parent (RL): $RL(\%)=\left(\frac{n_{lp}}{n}\right)\times 100$.

where S is the standard deviation and $\bar{X}$ is the mean of the F_1_ phenotype values, respectively. MP is the mid-parent value. hp is the number of F_1_ hybrids with phenotype values exceeding the high parent value. lp is the number of F_1_ hybrids with phenotype values below the low parent value. n is the total number of individuals in the F_1_ hybrid population.

### 4.5. Genetic Diversity Analysis

Genetic diversity is used to evaluate the quality of SNP markers. The polymorphism information content (PIC) for SNP loci was calculated using the formula [47]:

$$PIC=1-\sum_{i=1}^{n}p_{i}^{2}-\sum_{i=1}^{n-1}\sum_{j=i+1}^{n}2p_{i}^{2}p_{j}^{2}$$

where $p_{i}$, $p_{j}$ are the frequency of the i th or j th allele at a given SNP locus; n is the total number of alleles at this locus.

As biallelic SNPs, the number of effective alleles (Ne), observed heterozygosity (Ho), and expected heterozygosity (He) were calculated for moderately polymorphic loci (PIC ≥ 0.25) using the formulas:

$$N_{e}=1/\sum_{i=1}^{n}p_{i}^{2}$$

$$H_{o}=\frac{H}{N}$$

$$H_{e}=1-\sum_{i=1}^{n}p_{i}^{2}$$

where $p_{i}$ is the frequency of the i th allele at a given SNP locus; n is the total number of alleles at this locus. H is the number of heterozygous individuals within a population, and N is the total number of individuals.

For moderately polymorphic loci (PIC ≥ 0.25), Hardy–Weinberg equilibrium (HWE) *p*-value used the chi-square test to compare the observed genotype frequencies with the theoretically expected frequencies [48]:

$$\chi^{2}=\sum \frac{(O_{i}-E_{i}{)}^{2}}{E_{i}}\;(df=1)$$

where $O_{i}$ is the observed genotype frequency and $E_{i}$ is the heoretical expected genotype frequency, respectively. df is the degree of freedom and k is the number of genotype categories. For biallelic SNPs (k = 3 genotypes), *df* = 1.

### 4.6. Marker-Trait Association Analysis

To identify molecular markers associated with fruit weight variations, a significance test was performed for each marker locus as follows.

For every moderately polymorphic and HWE-conforming marker, individuals across the six populations were grouped according to their genotypes (e.g., AA, CA, CC). Prior to analysis, the normality of data within each genotypic group was assessed using the Shapiro–Wilk test and homogeneity of variances was tested using Levene’s test. For markers meeting both assumptions, a one-way analysis of variance (ANOVA) was used for loci with three or more genotypic groups, and an independent-samples t-test was used for loci with only two groups. For markers violating normality and/or homoscedasticity assumptions, non-parametric tests were employed: the Kruskal–Wallis H test for three or more groups, and the Mann–Whitney U test for two groups.

For markers where the one-way ANOVA yielded a significant result (*p* < 0.05), Tukey’s Honest Significant Difference (HSD) post hoc test was conducted to identify significantly genotypic pairs. A significant Kruskal–Wallis H test was followed by Dunn’s post hoc test with a Bonferroni adjustment for pairwise comparisons.

To account for the multiple comparisons performed across all marker loci, the false discovery rate was controlled using the Benjamini–Hochberg procedure. This correction was applied to all raw *p*-values generated from the initial tests, including ANOVA, t-test, Kruskal–Wallis, and Mann–Whitney U. An association was considered statistically significant at an FDR-adjusted *p*-value (q-value) of < 0.05. All phenotypic and genotypic data were organized in Microsoft Excel and analyzed with IBM SPSS Statistics 20.0. for statistical testing. All figures were created using OriginPro 2021.

### 4.7. Application of GAP

The accuracy of GS is typically measured by r between GEBV and true breeding values [40]. The accuracy of GAP is estimated by the correlation coefficient between GPV and OPV. This study employed the GAP model described by Shen et al. [9]. The genomic prediction value (GPV) was calculated as follows:

$$GPV=\alpha \times \left(Fx+\gamma \times \sum_{i=1}^{k}GnE+\mu \right)+\beta$$

where GnE is the genotype effects of the non-fixed-effect markers; Fx is the genotype effect fixed effect markers. k is the number of non-fixed-effect markers; μ is the phenotypic mean of the trait in the training population; γ is the shrinkage coefficient; α and β are linear regression coefficients and residual parameters, respectively.

## 5. Conclusions

Apple fruit weight is a polygenic trait; however, major genes controlling this trait may exist within specific genetic backgrounds. GAP, leveraging QTL-based markers, could effectively assist apple fruit size improvement within specific hybrid populations. Nevertheless, the evaluated 70 SNP markers exhibit population-specific applicability and are not generally effective across diverse apple hybrids. Given that the markers have a defined scope of application, achieving optimal prediction accuracy likely requires matching a specific marker combination with the genetic background of the target population. Applying a pre-screening strategy (PIC > 0.25, HWE-confirming, and MTA) to select suitable marker combinations can enhance prediction accuracy of GAP.

## Figures and Tables

**Table 1:** Genetic variations in fruit weight. CV: coefficient of variation; Ta: genetic transmitting ability of hybrids; H: heterosis; RH: ratio of higher than the high parent; RL: ratio of lower than low parent.

Hybrid Cross	Parent Fruit Weight (g)			F_1_ Population						
	Female Parent	Male Parent	Mid-Parent	Range (g)	Average (g)	CV (%)	Ta (%)	H (%)	RH (%)	RL (%)
‘Ralls Janet’ × ‘Ruiyang’	126.76	265.34	196.05	99.14–300.01	165.04	22.84%	84.18%	−15.82%	2.50%	8.75%
‘Ralls Janet’ × ‘YM1’	126.76	215.80	171.28	78.75–235.44	158.82	19.54%	92.72%	−7.28%	5.33%	17.33%
‘YM1’ × ‘Honeycrisp’	215.80	319.21	229.81	116.14–405.55	229.18	28.18%	85.91%	−14.09%	10.95%	45.26%
‘Ralls Janet’ × ‘Honeycrisp’	126.76	319.21	222.99	105.48–322.06	183.97	25.89%	82.50%	−17.50%	1.96%	5.88%
‘Ruixue’ × ‘Alps Otome’	271.16	38.00	154.58	110.75–376.72	223.76	21.55%	144.75%	44.75%	15.85%	0.00%
‘Fuping Qiuzi’ × ‘Ruixue’	30.75	271.16	190.96	13.60–99.14	39.72	44.11%	26.31%	−73.69%	0.00%	31.82%

**Table 2:** Pearson’s correlation coefficient (r) between genomic prediction value (GPV) and observed phenotypic value (OPV) for fruit weight in hybrid populations. Marker combinations used for GAP in each panel are listed in Table S5.

Hybrid Crosses	Marker Combination: Full Set; PIC > 0.25 and HWE-Conforming; MTA-Based.	r (GPV vs. OPV)
‘Ralls Janet’ × ‘Ruiyang’	70	−0.0713
	8	−0.0207
	1	0.2332 *
‘Ralls Janet’ × ‘YM1’	70	−0.0876
	13	−0.0976
	1	−0.2595 *
‘YM1’ × ‘Honeycrisp’	70	0.1152
	6	0.2035 *
	3	0.2475 *
‘Ralls Janet’ × ‘Honeycrisp’	70	0.1238
	7	0.1010
	2	0.1943
‘Ruixue’ × ‘Alps Otome’	70	0.0027
	13	0.0607
	0	NA
‘Fuping Qiuzi’ × ‘Ruixue’	70	0.4819 ***
	26	0.5755 ***
	10	0.5766 ***

Note: * indicates that the correlation between GPV and OPV is significant in the hybrid population (*p* < 0.05). *** indicates that the correlation between GPV and OPV is extremely significant in the hybrid population (*p* < 0.001). NA: Not applicable (the correlation of this locus was not calculated).

**Table 3:** Survey hybrid crosses and population sizes.

Hybrid Crosses	Number of Individuals
‘Ralls Janet’ × ‘Ruiyang’	80
‘Ralls Janet’ × ‘YM1’	75
‘YM1’ × ‘Honeycrisp’	137
‘Ralls Janet’ × ‘Honeycrisp’	113
‘Ruixue’ × ‘Alps Otome’	80
‘Fuping Qiuzi’ × ‘Ruixue’	88
Total	573

## Data Availability

The original contributions presented in this study are included in the article/Supplementary Material. Further inquiries can be directed to the corresponding author.

## Supplementary Materials

The following supporting information can be downloaded at: https://www.mdpi.com/article/10.3390/plants15010065/s1, Table S1: Phenotype data of six populations; Table S2: SNP genotyping results; Table S3: Genetic diversity analysis based on SNP genotyping results in six hybrid populations; Table S4: Significance of marker-trait association (MTA) for fruit weight in hybrid populations. Table S5. Marker combinations used for GAP analysis in each hybrid cross. Table S6. MTA-based SNPs for fruit weight were screened in six hybrid populations. Table S7. Significance tests of differences among genotypic groups were FDR-adjusted (False Discovery Rate).

## Abbreviations

The following abbreviations are used in this manuscript:

GAP	genome-assisted prediction
QTL	quantitative trait locus
MTA	marker-trait association
PIC	polymorphism information content
HWE	Hardy–Weinberg equilibrium
LG	Linkage Groups
GWAS	genome-wide association study
SNP	single nucleotide polymorphism
GS	Genomic selection
GEBV	genome estimated breeding values
GPV	Genomic Prediction Value
OPV	Observed Phenotypic Value
CV	coefficient of variation
H	heterosis
RL	Ratio of lower than low parent
RH	Ratio of higher than the high parent
Ta	genetic transmitting ability of hybrids
ANOVA	one-way analysis of variance
HSD	Tukey’s Honest Significant Difference
CTAB	cetyltrimethylammonium bromide
GBTS	Genotyping by Target Sequencing
FDR	False Discovery Rate
