# Supplementary material for: Application of Genome-Assisted Prediction (GAP) for Apple Fruit Weight
Source: Plants (Basel). 2025 Dec 25;15(1):65. doi: 10.3390/plants15010065 (PMC12787709; doi:10.3390/plants15010065)
Supplement: Supplementary file 1 [file plants-15-00065-s001.zip › Supplementary Table 3-7.-cleaned-up.pdf]

Supplementary Table 3. Genetic diversity analysis based on SNP genotyping results in six hybrid populations. Malus domestica Borkh. 'Ralls Janet' × 'Ruiyang', *Malus domestica* Borkh. 'Ralls Janet' × 'YM', Malus domestica Borkh. 'YM1' × 'Honeycrisp', Malus domestica Borkh. 'Ralls Janet' × 'Honeycrisp', Malus domestica Borkh. 'Ruixue' × 'Alps Otome', *Malus. Prunifolia* (Willd.) *Borkh.* 'Fuping Qiuzi' × 'Ruixue' Malus domestica Borkh.

Hybrid crosses	Markers	Polymorphism information content (PIC)	The number of effective alleles (Ne)	Observed heterozygosity (Ho)	Expected heterozygosity (He)	Hardy-Weinberg equilibrium (HWE) p-value
'Ralls Janet' × 'Ruiyang'	XDY157	0.0000	NA	NA	NA	NA
'Ralls Janet' × 'Ruiyang'	XDY160	0.0000	NA	NA	NA	NA
'Ralls Janet' × 'Ruiyang'	ddy6	0.0000	NA	NA	NA	NA
'Ralls Janet' × 'Ruiyang'	dyy88	0.3318	1.7241	0.6000	0.4200	0.0001
'Ralls Janet' × 'Ruiyang'	HB123	0.0361	NA	NA	NA	NA
'Ralls Janet' × 'Ruiyang'	newdy292	0.0000	NA	NA	NA	NA
'Ralls Janet' × 'Ruiyang'	SIZESIZE11309	0.0000	NA	NA	NA	NA
'Ralls Janet' × 'Ruiyang'	SIZE12710	0.0000	NA	NA	NA	NA
'Ralls Janet' × 'Ruiyang'	SIZE12448	0.0000	NA	NA	NA	NA
'Ralls Janet' × 'Ruiyang'	SIZE1348	0.2834	1.5193	0.4375	0.3418	0.0123
'Ralls Janet' × 'Ruiyang'	SIZE1413	0.3672	1.9406	0.4500	0.4847	0.5221
'Ralls Janet' × 'Ruiyang'	SIZE1609	0.0000	NA	NA	NA	NA
'Ralls Janet' × 'Ruiyang'	SIZE2020	0.0000	NA	NA	NA	NA
'Ralls Janet' × 'Ruiyang'	SIZE2250	0.3047	1.6000	0.5000	0.3750	0.0029
'Ralls Janet' × 'Ruiyang'	SIZE2270	0.0000	NA	NA	NA	NA
'Ralls Janet' × 'Ruiyang'	SIZE2365	0.0000	NA	NA	NA	NA
'Ralls Janet' × 'Ruiyang'	SIZE2888	0.2923	1.5517	0.4625	0.3555	0.0071

‘Ralls Janet’ × ‘Ruiyang’	SIZE3450	0.3346	1.7389	0.6125	0.4249	0.0001
‘Ralls Janet’ × ‘Ruiyang’	SIZE3790	0.0000	NA	NA	NA	NA
‘Ralls Janet’ × ‘Ruiyang’	SIZE4161	0.0000	NA	NA	NA	NA
‘Ralls Janet’ × ‘Ruiyang’	SIZE4250	0.2923	1.5517	0.4625	0.3555	0.0071
‘Ralls Janet’ × ‘Ruiyang’	SIZE4378	0.0000	NA	NA	NA	NA
‘Ralls Janet’ × ‘Ruiyang’	SIZE4457	0.0000	NA	NA	NA	NA
‘Ralls Janet’ × ‘Ruiyang’	SIZE4595	0.0000	NA	NA	NA	NA
‘Ralls Janet’ × ‘Ruiyang’	SIZE4849	0.0000	NA	NA	NA	NA
‘Ralls Janet’ × ‘Ruiyang’	SIZE4976	0.0000	NA	NA	NA	NA
‘Ralls Janet’ × ‘Ruiyang’	SIZE5929	0.0000	NA	NA	NA	NA
‘Ralls Janet’ × ‘Ruiyang’	SIZE7070	0.0000	NA	NA	NA	NA
‘Ralls Janet’ × ‘Ruiyang’	SIZE8111	0.0476	NA	NA	NA	NA
‘Ralls Janet’ × ‘Ruiyang’	SIZE8165	0.0476	NA	NA	NA	NA
‘Ralls Janet’ × ‘Ruiyang’	SIZE832	0.0587	NA	NA	NA	NA
‘Ralls Janet’ × ‘Ruiyang’	SIZE8397-02	0.2738	1.4868	0.4125	0.3274	0.0201
‘Ralls Janet’ × ‘Ruiyang’	SIZE8558	0.0000	NA	NA	NA	NA
‘Ralls Janet’ × ‘Ruiyang’	SIZE8646	0.0476	NA	NA	NA	NA
‘Ralls Janet’ × ‘Ruiyang’	SIZE8824	0.3750	1.9997	0.5875	0.4999	0.1171
‘Ralls Janet’ × ‘Ruiyang’	SIZE906	0.3702	1.9629	0.8625	0.4905	0.0000
‘Ralls Janet’ × ‘Ruiyang’	SIZE9100	0.0000	NA	NA	NA	NA
‘Ralls Janet’ × ‘Ruiyang’	SIZE9195	0.0000	NA	NA	NA	NA
‘Ralls Janet’ × ‘Ruiyang’	SIZE9471	0.3288	1.7092	0.5875	0.4149	0.0002

‘Ralls Janet’ × ‘Ruiyang’	SIZE9751	0.3288	1.7092	0.5375	0.4149	0.0082
‘Ralls Janet’ × ‘YM1’	XDY157	0.0000	NA	NA	NA	NA
‘Ralls Janet’ × ‘YM1’	XDY160	0.0000	NA	NA	NA	NA
‘Ralls Janet’ × ‘YM1’	ddy6	0.0000	NA	NA	NA	NA
‘Ralls Janet’ × ‘YM1’	dyy88	0.2793	1.5052	0.4267	0.3356	0.0188
‘Ralls Janet’ × ‘YM1’	HB123	0.2983	1.5743	0.4800	0.3648	0.0062
‘Ralls Janet’ × ‘YM1’	newdy292	0.0000	NA	NA	NA	NA
‘Ralls Janet’ × ‘YM1’	SIZE11309	0.0260	NA	NA	NA	NA
‘Ralls Janet’ × ‘YM1’	SIZE12710	0.0132	NA	NA	NA	NA
‘Ralls Janet’ × ‘YM1’	SIZE12448	0.0000	NA	NA	NA	NA
‘Ralls Janet’ × ‘YM1’	SIZE1348	0.2843	1.5225	0.4400	0.3432	0.0146
‘Ralls Janet’ × ‘YM1’	SIZE1413	0.3746	1.9968	0.4533	0.4992	0.4262
‘Ralls Janet’ × ‘YM1’	SIZE1609	0.0000	NA	NA	NA	NA
‘Ralls Janet’ × ‘YM1’	SIZE2020	0.0000	NA	NA	NA	NA
‘Ralls Janet’ × ‘YM1’	SIZE2250	0.0000	NA	NA	NA	NA
‘Ralls Janet’ × ‘YM1’	SIZE2270	0.0000	NA	NA	NA	NA
‘Ralls Janet’ × ‘YM1’	SIZE2365	0.0000	NA	NA	NA	NA
‘Ralls Janet’ × ‘YM1’	SIZE2888	0.0000	NA	NA	NA	NA
‘Ralls Janet’ × ‘YM1’	SIZE3450	0.3750	1.9996	0.5333	0.4999	0.5626
‘Ralls Janet’ × ‘YM1’	SIZE3790	0.0000	NA	NA	NA	NA
‘Ralls Janet’ × ‘YM1’	SIZE4161	0.0000	NA	NA	NA	NA
‘Ralls Janet’ × ‘YM1’	SIZE4250	0.3696	1.9579	0.5867	0.4892	0.0846

‘Ralls Janet’ × ‘YM1’	SIZE4378	0.0000	NA	NA	NA	NA
‘Ralls Janet’ × ‘YM1’	SIZE4457	0.0000	NA	NA	NA	NA
‘Ralls Janet’ × ‘YM1’	SIZE4595	0.3068	1.6085	0.5067	0.3783	0.0033
‘Ralls Janet’ × ‘YM1’	SIZE4849	0.0000	NA	NA	NA	NA
‘Ralls Janet’ × ‘YM1’	SIZE4976	0.0000	NA	NA	NA	NA
‘Ralls Janet’ × ‘YM1’	SIZE5929	0.0000	NA	NA	NA	NA
‘Ralls Janet’ × ‘YM1’	SIZE7070	0.0000	NA	NA	NA	NA
‘Ralls Janet’ × ‘YM1’	SIZE8111	0.2891	1.5398	0.4533	0.3506	0.0111
‘Ralls Janet’ × ‘YM1’	SIZE8165	0.2891	1.5398	0.4533	0.3506	0.0111
‘Ralls Janet’ × ‘YM1’	SIZE832	0.3748	1.9986	0.9733	0.4996	0.0000
‘Ralls Janet’ × ‘YM1’	SIZE8397-02	0.3746	1.9968	0.9600	0.4992	0.0000
‘Ralls Janet’ × ‘YM1’	SIZE8558	0.0132	NA	NA	NA	NA
‘Ralls Janet’ × ‘YM1’	SIZE8646	0.2688	1.4706	0.4000	0.3200	0.0304
‘Ralls Janet’ × ‘YM1’	SIZE8824	0.3748	1.9986	0.4933	0.4996	0.9129
‘Ralls Janet’ × ‘YM1’	SIZE906	0.3286	1.7082	0.5867	0.4146	0.0003
‘Ralls Janet’ × ‘YM1’	SIZE9100	0.0000	NA	NA	NA	NA
‘Ralls Janet’ × ‘YM1’	SIZE9195	0.0000	NA	NA	NA	NA
‘Ralls Janet’ × ‘YM1’	SIZE9471	0.2741	1.4879	0.4133	0.3279	0.0241
‘Ralls Janet’ × ‘YM1’	SIZE9751	0.3108	1.6255	0.5200	0.3848	0.0023
‘YM1' × ‘Honeycrisp'	XDY157	0.0000	NA	NA	NA	NA
‘YM1' × ‘Honeycrisp'	XDY160	0.0000	NA	NA	NA	NA
‘YM1' × ‘Honeycrisp'	ddy6	0.0000	NA	NA	NA	NA

‘YM1' × ‘Honeycrisp'	dyy88	0.0000	NA	NA	NA	NA
‘YM1' × ‘Honeycrisp'	HB123	0.3591	1.8840	0.5036	0.4692	0.3902
‘YM1' × ‘Honeycrisp'	newdy292	0.2888	1.5388	0.4526	0.3502	0.0006
‘YM1' × ‘Honeycrisp'	SIZE11309	0.2836	1.5199	0.4380	0.3421	0.0010
‘YM1' × ‘Honeycrisp'	SIZE12710	0.0000	NA	NA	NA	NA
‘YM1' × ‘Honeycrisp'	SIZE12448	0.0000	NA	NA	NA	NA
‘YM1' × ‘Honeycrisp'	SIZE1348	0.2862	1.5294	0.4453	0.3461	0.0008
‘YM1' × ‘Honeycrisp'	SIZE1413	0.3750	2.0000	0.5328	0.5000	0.4419
‘YM1' × ‘Honeycrisp'	SIZE1609	0.0000	NA	NA	NA	NA
‘YM1' × ‘Honeycrisp'	SIZE2020	0.0000	NA	NA	NA	NA
‘YM1' × ‘Honeycrisp'	SIZE2250	0.0000	NA	NA	NA	NA
‘YM1' × ‘Honeycrisp'	SIZE2270	0.0000	NA	NA	NA	NA
‘YM1' × ‘Honeycrisp'	SIZE2365	0.0000	NA	NA	NA	NA
‘YM1' × ‘Honeycrisp'	SIZE2888	0.0000	NA	NA	NA	NA
‘YM1' × ‘Honeycrisp'	SIZE3450	0.3750	2.0000	0.4453	0.5000	0.2000
‘YM1' × ‘Honeycrisp'	SIZE3790	0.0000	NA	NA	NA	NA
‘YM1' × ‘Honeycrisp'	SIZE4161	0.0000	NA	NA	NA	NA
‘YM1' × ‘Honeycrisp'	SIZE4250	0.3012	1.5860	0.4891	0.3695	0.0002
‘YM1' × ‘Honeycrisp'	SIZE4378	0.0000	NA	NA	NA	NA
‘YM1' × ‘Honeycrisp'	SIZE4457	0.0000	NA	NA	NA	NA
‘YM1' × ‘Honeycrisp'	SIZE4595	0.3145	1.6417	0.5328	0.3909	0.0000
‘YM1' × ‘Honeycrisp'	SIZE4849	0.0000	NA	NA	NA	NA

‘YM1' × ‘Honeycrisp'	SIZE4976	0.0000	NA	NA	NA	NA
‘YM1' × ‘Honeycrisp'	SIZE5929	0.0000	NA	NA	NA	NA
‘YM1' × ‘Honeycrisp'	SIZE7070	0.0000	NA	NA	NA	NA
‘YM1' × ‘Honeycrisp'	SIZE8111	0.3452	1.7974	0.5912	0.4436	0.0001
‘YM1' × ‘Honeycrisp'	SIZE8165	0.3331	1.7311	0.6058	0.4223	0.0000
‘YM1' × ‘Honeycrisp'	SIZE832	0.3058	1.6047	0.5036	0.3768	0.0001
‘YM1' × ‘Honeycrisp'	SIZE8397-02	0.0352	NA	NA	NA	NA
‘YM1' × ‘Honeycrisp'	SIZE8558	0.0000	NA	NA	NA	NA
‘YM1' × ‘Honeycrisp'	SIZE8646	0.3591	1.8840	0.3431	0.4692	0.0017
‘YM1' × ‘Honeycrisp'	SIZE8824	0.3747	1.9973	0.4818	0.4993	0.6802
‘YM1' × ‘Honeycrisp'	SIZE906	0.3750	1.9999	0.9927	0.5000	0.0000
‘YM1' × ‘Honeycrisp'	SIZE9100	0.0000	NA	NA	NA	NA
‘YM1' × ‘Honeycrisp'	SIZE9195	0.0000	NA	NA	NA	NA
‘YM1' × ‘Honeycrisp'	SIZE9471	0.2888	1.5388	0.4526	0.3502	0.0006
‘YM1' × ‘Honeycrisp'	SIZE9751	0.0072	NA	NA	NA	NA
‘Ralls Janet’ × ‘Honeycrisp’	XDY157	0.0000	NA	NA	NA	NA
‘Ralls Janet’ × ‘Honeycrisp’	XDY160	0.0000	NA	NA	NA	NA
‘Ralls Janet’ × ‘Honeycrisp’	ddy6	0.0000	NA	NA	NA	NA
‘Ralls Janet’ × ‘Honeycrisp’	dyy88	0.3236	1.6835	0.5664	0.4060	0.0000
‘Ralls Janet’ × ‘Honeycrisp’	HB123	0.2607	1.4454	0.3805	0.3081	0.0125
‘Ralls Janet’ × ‘Honeycrisp’	newdy292	0.2976	1.5716	0.4779	0.3637	0.0008
‘Ralls Janet’ × ‘Honeycrisp’	SIZE11309	0.2884	1.5372	0.4513	0.3495	0.0019

‘Ralls Janet’ × ‘Honeycrisp’	SIZE12710	0.0000	NA	NA	NA	NA
‘Ralls Janet’ × ‘Honeycrisp’	SIZE12448	0.0000	NA	NA	NA	NA
‘Ralls Janet’ × ‘Honeycrisp’	SIZE1348	0.0423	NA	NA	NA	NA
‘Ralls Janet’ × ‘Honeycrisp’	SIZE1413	0.3662	1.9332	0.5133	0.4827	0.5012
‘Ralls Janet’ × ‘Honeycrisp’	SIZE1609	0.0000	NA	NA	NA	NA
‘Ralls Janet’ × ‘Honeycrisp’	SIZE2020	0.0000	NA	NA	NA	NA
‘Ralls Janet’ × ‘Honeycrisp’	SIZE2250	0.0259	NA	NA	NA	NA
‘Ralls Janet’ × ‘Honeycrisp’	SIZE2270	0.0000	NA	NA	NA	NA
‘Ralls Janet’ × ‘Honeycrisp’	SIZE2365	0.0000	NA	NA	NA	NA
‘Ralls Janet’ × ‘Honeycrisp’	SIZE2888	0.0259	NA	NA	NA	NA
‘Ralls Janet’ × ‘Honeycrisp’	SIZE3450	0.3747	1.9975	0.5221	0.4994	0.6282
‘Ralls Janet’ × ‘Honeycrisp’	SIZE3790	0.0000	NA	NA	NA	NA
‘Ralls Janet’ × ‘Honeycrisp’	SIZE4161	0.0000	NA	NA	NA	NA
‘Ralls Janet’ × ‘Honeycrisp’	SIZE4250	0.2976	1.5716	0.4779	0.3637	0.0008
‘Ralls Janet’ × ‘Honeycrisp’	SIZE4378	0.0088	NA	NA	NA	NA
‘Ralls Janet’ × ‘Honeycrisp’	SIZE4457	0.0000	NA	NA	NA	NA
‘Ralls Janet’ × ‘Honeycrisp’	SIZE4595	0.0000	NA	NA	NA	NA
‘Ralls Janet’ × ‘Honeycrisp’	SIZE4849	0.0000	NA	NA	NA	NA
‘Ralls Janet’ × ‘Honeycrisp’	SIZE4976	0.0000	NA	NA	NA	NA
‘Ralls Janet’ × ‘Honeycrisp’	SIZE5929	0.0000	NA	NA	NA	NA
‘Ralls Janet’ × ‘Honeycrisp’	SIZE7070	0.0000	NA	NA	NA	NA
‘Ralls Janet’ × ‘Honeycrisp’	SIZE8111	0.0342	NA	NA	NA	NA

‘Ralls Janet’ × ‘Honeycrisp’	SIZE8165	0.0342	NA	NA	NA	NA
‘Ralls Janet’ × ‘Honeycrisp’	SIZE832	0.2819	1.5143	0.4336	0.3396	0.0033
‘Ralls Janet’ × ‘Honeycrisp’	SIZE8397-02	0.3748	1.9986	0.9735	0.4996	0.0000
‘Ralls Janet’ × ‘Honeycrisp’	SIZE8558	0.0000	NA	NA	NA	NA
‘Ralls Janet’ × ‘Honeycrisp’	SIZE8646	0.0259	NA	NA	NA	NA
‘Ralls Janet’ × ‘Honeycrisp’	SIZE8824	0.3750	1.9998	0.5310	0.5000	0.5096
‘Ralls Janet’ × ‘Honeycrisp’	SIZE906	0.3750	1.9998	0.9912	0.5000	0.0000
‘Ralls Janet’ × ‘Honeycrisp’	SIZE9100	0.0000	NA	NA	NA	NA
‘Ralls Janet’ × ‘Honeycrisp’	SIZE9195	0.0000	NA	NA	NA	NA
‘Ralls Janet’ × ‘Honeycrisp’	SIZE9471	0.3747	1.9975	0.6283	0.4994	0.0061
‘Ralls Janet’ × ‘Honeycrisp’	SIZE9751	0.3189	1.6615	0.5310	0.3982	0.0004
‘Ruixue’ × ‘Alps Otome’	XDY157	0.0000	NA	NA	NA	NA
‘Ruixue’ × ‘Alps Otome’	XDY160	0.0000	NA	NA	NA	NA
‘Ruixue’ × ‘Alps Otome’	ddy6	0.0000	NA	NA	NA	NA
‘Ruixue’ × ‘Alps Otome’	dyy88	0.0123	NA	NA	NA	NA
‘Ruixue’ × ‘Alps Otome’	HB123	0.0885	NA	NA	NA	NA
‘Ruixue’ × ‘Alps Otome’	newdy292	0.2966	1.5679	0.4750	0.3622	0.0053
‘Ruixue’ × ‘Alps Otome’	SIZE11309	0.3007	1.5840	0.4875	0.3687	0.0039
‘Ruixue’ × ‘Alps Otome’	SIZE12710	0.0000	NA	NA	NA	NA
‘Ruixue’ × ‘Alps Otome’	SIZE12448	0.0000	NA	NA	NA	NA
‘Ruixue’ × ‘Alps Otome’	SIZE1348	0.3047	1.6000	0.5000	0.3750	0.0025
‘Ruixue’ × ‘Alps Otome’	SIZE1413	0.1984	NA	NA	NA	NA

‘Ruixue’ × ‘Alps Otome’	SIZE1609	0.0000	NA	NA	NA	NA
‘Ruixue’ × ‘Alps Otome’	SIZE2020	0.0000	NA	NA	NA	NA
‘Ruixue’ × ‘Alps Otome’	SIZE2250	0.2879	1.5355	0.4500	0.3488	0.0094
‘Ruixue’ × ‘Alps Otome’	SIZE2270	0.0000	NA	NA	NA	NA
‘Ruixue’ × ‘Alps Otome’	SIZE2365	0.0000	NA	NA	NA	NA
‘Ruixue’ × ‘Alps Otome’	SIZE2888	0.2688	1.4706	0.4000	0.3200	0.0253
‘Ruixue’ × ‘Alps Otome’	SIZE3450	0.3693	1.9560	0.5500	0.4888	0.2623
‘Ruixue’ × ‘Alps Otome’	SIZE3790	0.0000	NA	NA	NA	NA
‘Ruixue’ × ‘Alps Otome’	SIZE4161	0.0000	NA	NA	NA	NA
‘Ruixue’ × ‘Alps Otome’	SIZE4250	0.3253	1.6918	0.5000	0.4089	0.0437
‘Ruixue’ × ‘Alps Otome’	SIZE4378	0.1763	NA	NA	NA	NA
‘Ruixue’ × ‘Alps Otome’	SIZE4457	0.1839	NA	NA	NA	NA
‘Ruixue’ × ‘Alps Otome’	SIZE4595	0.3640	1.9176	0.4512	0.4785	0.6055
‘Ruixue’ × ‘Alps Otome’	SIZE4849	0.0000	NA	NA	NA	NA
‘Ruixue’ × ‘Alps Otome’	SIZE4976	0.0000	NA	NA	NA	NA
‘Ruixue’ × ‘Alps Otome’	SIZE5929	0.0000	NA	NA	NA	NA
‘Ruixue’ × ‘Alps Otome’	SIZE7070	0.0000	NA	NA	NA	NA
‘Ruixue’ × ‘Alps Otome’	SIZE8111	0.0885	NA	NA	NA	NA
‘Ruixue’ × ‘Alps Otome’	SIZE8165	0.0784	NA	NA	NA	NA
‘Ruixue’ × ‘Alps Otome’	SIZE832	0.3367	1.7499	0.4512	0.4285	0.6318
‘Ruixue’ × ‘Alps Otome’	SIZE8397-02	0.3340	1.7357	0.6098	0.4239	0.0001
‘Ruixue’ × ‘Alps Otome’	SIZE8558	0.3312	1.7212	0.5976	0.4190	0.0001

‘Ruixue’ × ‘Alps Otome’	SIZE8646	0.3741	1.9926	0.5488	0.4981	0.3573
‘Ruixue’ × ‘Alps Otome’	SIZE8824	0.3418	1.7777	0.5488	0.4375	0.0212
‘Ruixue’ × ‘Alps Otome’	SIZE906	0.3627	1.9081	0.3415	0.4759	0.0105
‘Ruixue’ × ‘Alps Otome’	SIZE9100	0.0000	NA	NA	NA	NA
‘Ruixue’ × ‘Alps Otome’	SIZE9195	0.0000	NA	NA	NA	NA
‘Ruixue’ × ‘Alps Otome’	SIZE9471	0.3007	1.5840	0.4875	0.3687	0.0039
‘Ruixue’ × ‘Alps Otome’	SIZE9751	0.3750	1.9997	0.9872	0.4999	0.0000
‘Fuping Qiuzi’ × ‘Ruixue’	XDY157	0.3082	1.6145	0.5114	0.3806	0.0013
‘Fuping Qiuzi’ × ‘Ruixue’	XDY160	0.3082	1.6145	0.5114	0.3806	0.0013
‘Fuping Qiuzi’ × ‘Ruixue’	ddy6	0.3082	1.6145	0.5114	0.3806	0.0013
‘Fuping Qiuzi’ × ‘Ruixue’	dyy88	0.0000	NA	NA	NA	NA
‘Fuping Qiuzi’ × ‘Ruixue’	HB123	0.0000	NA	NA	NA	NA
‘Fuping Qiuzi’ × ‘Ruixue’	newdy292	0.0000	NA	NA	NA	NA
‘Fuping Qiuzi’ × ‘Ruixue’	SIZE11309	0.3011	1.5854	0.4886	0.3693	0.002
‘Fuping Qiuzi’ × ‘Ruixue’	SIZE12710	0.0000	NA	NA	NA	NA
‘Fuping Qiuzi’ × ‘Ruixue’	SIZE12448	0.3047	1.6000	0.5000	0.3750	0.002
‘Fuping Qiuzi’ × ‘Ruixue’	SIZE1348	0.0000	NA	NA	NA	NA
‘Fuping Qiuzi’ × ‘Ruixue’	SIZE1413	0.3047	1.6000	0.5000	0.3750	0.002
‘Fuping Qiuzi’ × ‘Ruixue’	SIZE1609	0.3082	1.6145	0.5114	0.3806	0.0013
‘Fuping Qiuzi’ × ‘Ruixue’	SIZE2020	0.2896	1.5414	0.4545	0.3512	0.006
‘Fuping Qiuzi’ × ‘Ruixue’	SIZE2250	0.2896	1.5414	0.4545	0.3512	0.006
‘Fuping Qiuzi’ × ‘Ruixue’	SIZE2270	0.3011	1.5854	0.4886	0.3693	0.002

‘Fuping Qiuzi’ × ‘Ruixue’	SIZE2365	0.2935	1.5561	0.4659	0.3574	0.004
‘Fuping Qiuzi’ × ‘Ruixue’	SIZE2888	0.0000	NA	NA	NA	NA
‘Fuping Qiuzi’ × ‘Ruixue’	SIZE3450	0.3655	1.9280	0.4659	0.4813	0.764
‘Fuping Qiuzi’ × ‘Ruixue’	SIZE3790	0.3011	1.5854	0.4886	0.3693	0.002
‘Fuping Qiuzi’ × ‘Ruixue’	SIZE4161	0.3047	1.6000	0.5000	0.3750	0.002
‘Fuping Qiuzi’ × ‘Ruixue’	SIZE4250	0.3745	1.9959	0.4318	0.4990	0.207
‘Fuping Qiuzi’ × ‘Ruixue’	SIZE4378	0.0000	NA	NA	NA	NA
‘Fuping Qiuzi’ × ‘Ruixue’	SIZE4457	0.3297	NA	NA	NA	NA
‘Fuping Qiuzi’ × ‘Ruixue’	SIZE4595	0.3047	1.6000	0.5000	0.3750	0.002
‘Fuping Qiuzi’ × ‘Ruixue’	SIZE4849	0.2679	1.4676	0.3977	0.3186	0.020
‘Fuping Qiuzi’ × ‘Ruixue’	SIZE4976	0.2813	1.5119	0.4318	0.3386	0.010
‘Fuping Qiuzi’ × ‘Ruixue’	SIZE5929	0.3047	1.6000	0.5000	0.3750	0.002
‘Fuping Qiuzi’ × ‘Ruixue’	SIZE7070	0.2974	1.5708	0.4773	0.3634	0.003
‘Fuping Qiuzi’ × ‘Ruixue’	SIZE8111	0.3374	NA	NA	NA	NA
‘Fuping Qiuzi’ × ‘Ruixue’	SIZE8165	0.0222	NA	NA	NA	NA
‘Fuping Qiuzi’ × ‘Ruixue’	SIZE832	0.3211	1.6717	0.5341	0.4018	0.002
‘Fuping Qiuzi’ × ‘Ruixue’	SIZE8397-02	0.3269	1.6996	0.5795	0.4116	0.000
‘Fuping Qiuzi’ × ‘Ruixue’	SIZE8558	0.3082	1.6145	0.5114	0.3806	0.001
‘Fuping Qiuzi’ × ‘Ruixue’	SIZE8646	0.3703	NA	NA	NA	NA
‘Fuping Qiuzi’ × ‘Ruixue’	SIZE8824	0.2953	1.5630	0.4713	0.3602	0.004
‘Fuping Qiuzi’ × ‘Ruixue’	SIZE906	0.3589	NA	NA	NA	NA
‘Fuping Qiuzi’ × ‘Ruixue’	SIZE9100	0.2679	1.4676	0.3977	0.3186	0.020

‘Fuping Qiuzi’ × ‘Ruixue’	SIZE9195	0.2896	1.5414	0.4545	0.3512	0.006
‘Fuping Qiuzi’ × ‘Ruixue’	SIZE9471	0.0000	NA	NA	NA	NA
‘Fuping Qiuzi’ × ‘Ruixue’	SIZE9751	0.3729	1.9836	0.5000	0.4959	0.9377

Note: NA is not applicable (the value was not calculated because of low polymorphism in this loci).

Supplementary Table 4. Significance of marker-trait association (MTA) for fruit weight in hybrid populations. ‘Ralls Janet’ × ‘Ruiyang’, ‘Ralls Janet’ × ‘YM1’, ‘YM1’ × ‘Honeycrisp’, ‘Ralls Janet’ × ‘Honeycrisp’, ‘Ruixue’ × ‘Alps Otome’, ‘Fuping Qiuzi’ × ‘Ruixue’.

Markers	Hybrid crosses	Genetype	Number	Apple fruit weight(M±SD)
SIZE1348	‘Ralls Janet’ × ‘Ruiyang’	AA	45	163.58±35.63a
		AT	35	166.83±40.83a
SIZE1413	‘Ralls Janet’ × ‘Ruiyang’	CC	29	164.68±33.01a
		CT	36	168.83±40.74a
		TT	15	156.45±40.09a
SIZE2250	‘Ralls Janet’ × ‘Ruiyang’	TA	40	156.25±34.10a
		TT	40	173.76±39.63b
SIZE2888	‘Ralls Janet’ × ‘Ruiyang’	GG	43	161.70±32.94a
		GT	37	168.84±42.86a
SIZE4250	‘Ralls Janet’ × ‘Ruiyang’	AA	43	166.16±32.04a
		AG	37	163.65±43.93a
SIZE8397-02	‘Ralls Janet’ × ‘Ruiyang’	AG	33	174.29±35.30a
		GG	47	158.48±38.45a
SIZE8824	‘Ralls Janet’ × ‘Ruiyang’	GG	17	162.00±29.04a
		GT	47	170.07±42.95a
		TT	16	153.30±26.83a
SIZE9751	‘Ralls Janet’ × ‘Ruiyang’	AA	2	/
		CA	43	173.24±36.52a
		CC	35	156.60±38.08a
dyy88	‘Ralls Janet’ × ‘YM1’	TC	32	157.22±32.25a
		TT	43	160.01±30.44a
HB123	‘Ralls Janet’ × ‘YM1’	TC	36	161.70±28.59a
		TT	39	156.16±33.29a
SIZE1348	‘Ralls Janet’ × ‘YM1’	AA	42	163.06±31.74a
		AT	33	153.42±29.73a
SIZE1413	‘Ralls Janet’ × ‘YM1’	CC	22	153.24±31.08a
		CT	34	163.15±32.64a
		TT	19	157.53±28.39a
SIZE3450	‘Ralls Janet’ × ‘YM1’	AA	18	153.73±33.64a
		TA	40	158.07±30.74a
		TT	17	165.97±29.41a
SIZE4250	‘Ralls Janet’ × ‘YM1’	AA	21	164.50±29.21a
		AG	44	157.14±28.62a
		GG	10	154.28±44.70a
SIZE4595	‘Ralls Janet’ × ‘YM1’	AA	37	150.71±31.25a
		AC	38	166.72±29.10b
SIZE8111	‘Ralls Janet’ × ‘YM1’	GA	34	156.88±28.58a
		GG	41	160.43±33.20a

SIZE8165	'Ralls Janet' × 'YM1'	TC	34	159.56±26.39a
		TT	41	158.21±34.74a
SIZE8646	'Ralls Janet' × 'YM1'	CT	30	159.86±30.85a
		TT	45	158.12±31.49a
SIZE8824	'Ralls Janet' × 'YM1'	GG	18	166.52±31.92a
		GT	37	156.86±30.12a
		TT	20	155.51±32.34a
SIZE9471	'Ralls Janet' × 'YM1'	AA	44	161.73±32.16a
		AG	31	154.69±29.40a
SIZE9751	'Ralls Janet' × 'YM1'	AA	36	160.06±28.34a
		CA	39	157.67±33.67a
HB123	'YM1' × 'Honeycrisp'	TC	69	220.18±60.59a
		TT	51	238.15±72.15a
SIZE11309	'YM1' × 'Honeycrisp'	CC	17	243.90±54.52a
		TA	60	217.38±57.22a
		TT	77	239.50±68.91b
SIZE1413	'YM1' × 'Honeycrisp'	TT	32	221.70±60.98a
		CT	73	230.13±64.50a
		CC	32	237.20±70.00a
SIZE3450	'YM1' × 'Honeycrisp'	TA	61	238.37±68.47a
		TT	38	203.08±54.13b
		AA	38	242.81±62.13a
SIZE8646	'YM1' × 'Honeycrisp'	CC	28	233.03±60.68a
		TT	62	221.89±64.49a
		CT	47	238.35±67.53a
SIZE8824	'YM1' × 'Honeycrisp'	GG	33	213.37±58.73b
		GT	66	228.76±64.63ab
		TT	38	245.91±67.74a
HB123	'Ralls Janet' × 'Honeycrisp'	TC	43	186.88±60.73a
		TT	70	172.60±40.97a
SIZE11309	'Ralls Janet' × 'Honeycrisp'	TA	51	165.23±49.53a
		TT	62	188.57±47.61b
SIZE1413	'Ralls Janet' × 'Honeycrisp'	CC	38	185.95±60.02a
		CT	58	170.77±45.51a
		TT	17	185.13±34.01a
SIZE3450	'Ralls Janet' × 'Honeycrisp'	AA	29	209.60±43.09a
		TA	59	177.99±48.06b
		TT	25	141.51±34.14c
SIZE832	'Ralls Janet' × 'Honeycrisp'	CC	64	173.26±49.05a
		GC	49	184.28±50.25a
SIZE8824	'Ralls Janet' × 'Honeycrisp'	GG	27	189.66±46.81a
		GT	60	171.31±47.56a
		TT	26	181.49±56.24a
SIZE9471	'Ralls Janet' × 'Honeycrisp'	AA	23	177.89±44.70a

		AG	71	181.94±51.69a
		GG	19	163.60±47.22a
		CC	16	210.63±24.72a
SIZE8646	‘Ruixue’ × ‘Alps Otome’	CT	45	227.28±54.96a
		TT	19	232.13±43.59a
		GG	33	217.48±48.99a
SIZE8824	‘Ruixue’ × ‘Alps Otome’	GT	45	229.02±49.63a
		TT	2	217.97±13.44a
SIZE1348	‘Ruixue’ × ‘Alps Otome’	AA	41	228.56±56.85a
		AT	39	217.84±38.00a
		CC	38	224.74±51.33a
SIZE832	‘Ruixue’ × ‘Alps Otome’	GC	37	220.31±46.81a
		GG	5	233.83±47.85a
		CC	18	235.74±53.90a
SIZE906	‘Ruixue’ × ‘Alps Otome’	CT	28	221.99±38.60a
		TT	34	216.56±52.28a
SIZE4250	‘Ruixue’ × ‘Alps Otome’	AA	38	225.56±46.52a
		AG	41	222.68±46.62a
		AA	31	224.14±47.06a
SIZE4595	‘Ruixue’ × ‘Alps Otome’	AC	37	223.48±46.37a
		CC	12	226.44±63.09a
SIZE2250	‘Ruixue’ × ‘Alps Otome’	TA	36	224.22±49.01a
		TT	44	224.16±49.06a
SIZE2888	‘Ruixue’ × ‘Alps Otome’	GG	48	222.40±45.93a
		GT	32	226.87±53.28a
SIZE9471	‘Ruixue’ × ‘Alps Otome’	AA	51	222.94±49.98a
		AG	29	226.39±47.22a
SIZE11309	‘Ruixue’ × ‘Alps Otome’	TA	39	230.10±43.97a
		TT	41	218.57±50.27a
		AA	12	220.68±54.30a
SIZE3450	‘Ruixue’ × ‘Alps Otome’	TA	44	227.78±45.63a
		TT	24	219.36±52.87a
newdy292	‘Ruixue’ × ‘Alps Otome’	TC	38	225.83±40.96a
		TT	42	222.70±55.28a
XDY157	‘Fuping Qiuzi’ × ‘Ruixue’	TG	45	36.54±15.93a
		TT	43	45.38±28.84a
XDY160	‘Fuping Qiuzi’ × ‘Ruixue’	GA	45	36.54±15.93a
		GG	43	45.38±28.84a
ddy6	‘Fuping Qiuzi’ × ‘Ruixue’	GA	45	33.89±11.26a
		GG	43	48.15±29.98b
SIZE11309	‘Fuping Qiuzi’ × ‘Ruixue’	TA	43	37.41±16.29a
		TT	45	44.15±28.47a
SIZE12448	‘Fuping Qiuzi’ × ‘Ruixue’	CA	44	34.04±11.34a
		CC	44	47.67±29.80b

SIZE1413	'Fuping Qiuzi' × 'Ruixue'	CC	44	43.26±28.98a
		CT	44	38.46±16.13a
SIZE4976	'Fuping Qiuzi' × 'Ruixue'	AA	50	46.69±29.04a
		AC	38	33.19±8.30b
SIZE2020	'Fuping Qiuzi' × 'Ruixue'	CC	48	47.57±29.24a
		CT	40	32.80±8.55b
SIZE9195	'Fuping Qiuzi' × 'Ruixue'	AA	48	49.11±28.49a
		AG	40	30.96±7.97b
SIZE2250	'Fuping Qiuzi' × 'Ruixue'	TA	40	30.92±7.99a
		TT	48	49.14±28.46b
SIZE2270	'Fuping Qiuzi' × 'Ruixue'	GA	43	30.92±8.50a
		GG	45	50.35±28.79b
SIZE2365	'Fuping Qiuzi' × 'Ruixue'	CC	47	49.78±28.31a
		CG	41	30.64±8.55b
SIZE1609	'Fuping Qiuzi' × 'Ruixue'	AA	43	44.13±28.87a
		GA	45	37.73±16.44a
SIZE3450	'Fuping Qiuzi' × 'Ruixue'	AA	15	47.67±42.79a
		TA	41	39.94±16.38a
		TT	32	38.85±18.47a
SIZE3790	'Fuping Qiuzi' × 'Ruixue'	TC	43	34.01±11.47a
		TT	45	47.40±29.52b
SIZE4161	'Fuping Qiuzi' × 'Ruixue'	AA	44	47.67±29.80a
		AG	44	34.04±11.34b
SIZE4250	'Fuping Qiuzi' × 'Ruixue'	AA	23	36.08±15.57a
		AG	38	39.24±17.12a
		GG	27	47.21±33.92a
SIZE4595	'Fuping Qiuzi' × 'Ruixue'	AC	44	41.33±17.30a
		CC	44	40.39±28.49a
SIZE4849	'Fuping Qiuzi' × 'Ruixue'	TG	35	34.94±14.66a
		TT	53	44.77±27.19a
SIZE5929	'Fuping Qiuzi' × 'Ruixue'	CC	44	45.15±28.54a
		CT	44	36.57±16.11a
SIZE7070	'Fuping Qiuzi' × 'Ruixue'	AA	46	43.33±28.16a
		AC	42	38.15±16.78a
SIZE832	'Fuping Qiuzi' × 'Ruixue'	CC	40	40.92±28.63a
		GC	47	39.57±16.39a
		GG	1	/
SIZE8558	'Fuping Qiuzi' × 'Ruixue'	CC	43	43.71±30.28a
		TC	45	38.13±13.98a
SIZE8824	'Fuping Qiuzi' × 'Ruixue'	GA	1	/
		GG	46	37.76±17.01a
		GT	41	44.47±29.06a
SIZE9100	'Fuping Qiuzi' × 'Ruixue'	GG	53	44.77±27.19a
		GT	35	34.94±14.66a

		AA	26	41.12±10.19a
SIZE9751	‘Fuping Qiuzi’ × ‘Ruixue’	CA	44	44.56±30.85a
		CC	18	31.44±11.07a

Note: / is discarded because the number of this genotype is less than 3 and analysis of variance cannot be conducted.

Supplementary Table 5. Marker combinations used for GAP analysis in each hybrid cross:(i) the full 70-marker set, (ii) the PIC > 0.25 & HWE-conforming subset, and (iii) the MTA-based subset, together with the exact markers comprising each combination.

Hybrid crosses	Marker combination		
	Full set;	PIC>0.25 and HWE-conforming;	MTA-based
‘Ralls Janet’ × ‘Ruiyang’	XDY127、XDY157、XDY160、XDY346、ddy6、dyy88、FDY190、HB123、newdy292、SIZE10554、SIZE10597、SIZE11309、SIZE11912、SIZE12448、SIZE12710、SIZE1348、SIZE1413、SIZE4976、SIZE2020、SIZE5112、SIZE9195、SIZE2250、SIZE2270、SIZE2365、SIZE2614、SIZE2733、SIZE2805、SIZE2888、SIZE2987、SIZE1609、SIZE273、SIZE6268、SIZE3067、SIZE5253、SIZE3158、SIZE3450、SIZE3790、SIZE4161、SIZE4250、SIZE4378、SIZE4457、SIZE4595、SIZE4849、SIZE5388、SIZE5464、SIZE5769、SIZE5929、SIZE6076、SIZE6459、SIZE6675、SIZE6815、SIZE6840、SIZE7016、SIZE7070、SIZE7418、SIZE7516、SIZE7624、SIZE8111、SIZE8165、SIZE8286、SIZE832、SIZE8397-02、SIZE8558、SIZE8646、SIZE8824、SIZE906、SIZE9100、SIZE9364、SIZE9471、SIZE9751	SIZE1348、SIZE1413、SIZE2250、SIZE2888、SIZE4250、SIZE8397-02、SIZE8824、SIZE9751	SIZE2250

‘Ralls Janet’ × ‘YM1’	XDY127、XDY157、XDY160、XDY346、ddy6、dyy88、FDY190、HB123、newdy292、SIZE10554、SIZE10597、SIZE11309、SIZE11912、SIZE12448、SIZE12710、SIZE1348、SIZE1413、SIZE4976、SIZE2020、SIZE5112、SIZE9195、SIZE2250、SIZE2270、SIZE2365、SIZE2614、SIZE2733、SIZE2805、SIZE2888、SIZE2987、SIZE1609、SIZE273、SIZE6268、SIZE3067、SIZE5253、SIZE3158、SIZE3450、SIZE3790、SIZE4161、SIZE4250、SIZE4378、SIZE4457、SIZE4595、SIZE4849、SIZE5388、SIZE5464、SIZE5769、SIZE5929、SIZE6076、SIZE6459、SIZE6675、SIZE6815、SIZE6840、SIZE7016、SIZE7070、SIZE7418、SIZE7516、SIZE7624、SIZE8111、SIZE8165、SIZE8286、SIZE832、SIZE8397-02、SIZE8558、SIZE8646、SIZE8824、SIZE906、SIZE9100、SIZE9364、SIZE9471、SIZE9751	dyy88、HB123、SIZE1348、SIZE1413、SIZE3450、SIZE4250、SIZE4595、SIZE8111、SIZE8165、SIZE8646、SIZE8824、SIZE9471、SIZE9751	SIZE4595
‘YM1’ × ‘Honeycrisp’	XDY127、XDY157、XDY160、XDY346、ddy6、dyy88、FDY190、HB123、newdy292、SIZE10554、SIZE10597、SIZE11309、SIZE11912、SIZE12448、SIZE12710、SIZE1348、SIZE1413、SIZE4976、SIZE2020、SIZE5112、SIZE9195、SIZE2250、SIZE2270、SIZE2365、SIZE2614、SIZE2733、SIZE2805、SIZE2888、SIZE2987、SIZE1609、SIZE273、SIZE6268、SIZE3067、SIZE5253、SIZE3158、SIZE3450、SIZE3790、SIZE4161、SIZE4250、SIZE4378、SIZE4457、SIZE4595、SIZE4849、SIZE5388、SIZE5464、SIZE5769、SIZE5929、SIZE6076、SIZE6459、SIZE6675、SIZE6815、SIZE6840、SIZE7016、SIZE7070、SIZE7418、SIZE7516、SIZE7624、SIZE8111、SIZE8165、SIZE8286、SIZE832、SIZE8397-02、SIZE8558、SIZE8646、SIZE8824、SIZE906、SIZE9100、SIZE9364、SIZE9471、SIZE9751	HB123、SIZE11309、SIZE1413、SIZE3450、SIZE8646、SIZE8824	SIZE11309、SIZE3450、SIZE8824

‘Ralls Janet’ × ‘Honeycrisp’	XDY127、XDY157、XDY160、XDY346、ddy6、dyy88、FDY190、HB123、newdy292、SIZE10554、SIZE10597、SIZE11309、SIZE11912、SIZE12448、SIZE12710、SIZE1348、SIZE1413、SIZE4976、SIZE2020、SIZE5112、SIZE9195、SIZE2250、SIZE2270、SIZE2365、SIZE2614、SIZE2733、SIZE2805、SIZE2888、SIZE2987、SIZE1609、SIZE273、SIZE6268、SIZE3067、SIZE5253、SIZE3158、SIZE3450、SIZE3790、SIZE4161、SIZE4250、SIZE4378、SIZE4457、SIZE4595、SIZE4849、SIZE5388、SIZE5464、SIZE5769、SIZE5929、SIZE6076、SIZE6459、SIZE6675、SIZE6815、SIZE6840、SIZE7016、SIZE7070、SIZE7418、SIZE7516、SIZE7624、SIZE8111、SIZE8165、SIZE8286、SIZE832、SIZE8397-02、SIZE8558、SIZE8646、SIZE8824、SIZE906、SIZE9100、SIZE9364、SIZE9471、SIZE9751	HB123、SIZE11309、SIZE1413、SIZE3450、SIZE832、SIZE8824、SIZE9471	SIZE11309、SIZE3450
‘Ruixue’ × ‘Alps Otome’	XDY127、XDY157、XDY160、XDY346、ddy6、dyy88、FDY190、HB123、newdy292、SIZE10554、SIZE10597、SIZE11309、SIZE11912、SIZE12448、SIZE12710、SIZE1348、SIZE1413、SIZE4976、SIZE2020、SIZE5112、SIZE9195、SIZE2250、SIZE2270、SIZE2365、SIZE2614、SIZE2733、SIZE2805、SIZE2888、SIZE2987、SIZE1609、SIZE273、SIZE6268、SIZE3067、SIZE5253、SIZE3158、SIZE3450、SIZE3790、SIZE4161、SIZE4250、SIZE4378、SIZE4457、SIZE4595、SIZE4849、SIZE5388、SIZE5464、SIZE5769、SIZE5929、SIZE6076、SIZE6459、SIZE6675、SIZE6815、SIZE6840、SIZE7016、SIZE7070、SIZE7418、SIZE7516、SIZE7624、SIZE8111、SIZE8165、SIZE8286、SIZE832、SIZE8397-02、SIZE8558、SIZE8646、SIZE8824、SIZE906、SIZE9100、SIZE9364、SIZE9471、SIZE9751	SIZE8646、SIZE8824、SIZE1348、SIZE832、SIZE906、SIZE4250 、SIZE4595 、SIZE2250 、SIZE2888 、SIZE9471 、SIZE11309、SIZE3450、newdy292	/

‘Fuping Qiuzi’ × ‘Ruixue’	XDY127、XDY157、XDY160、XDY346、ddy6、dyy88、FDY190、HB123、newdy292、SIZE10554、SIZE10597、SIZE11309、SIZE11912、SIZE12448、SIZE12710、SIZE1348、SIZE1413、SIZE4976、SIZE2020、SIZE5112、SIZE9195、SIZE2250、SIZE2270、SIZE2365、SIZE2614、SIZE2733、SIZE2805、SIZE2888、SIZE2987、SIZE1609、SIZE273、SIZE6268、SIZE3067、SIZE5253、SIZE3158、SIZE3450、SIZE3790、SIZE4161、SIZE4250、SIZE4378、SIZE4457、SIZE4595、SIZE4849、SIZE5388、SIZE5464、SIZE5769、SIZE5929、SIZE6076、SIZE6459、SIZE6675、SIZE6815、SIZE6840、SIZE7016、SIZE7070、SIZE7418、SIZE7516、SIZE7624、SIZE8111、SIZE8165、SIZE8286、SIZE832、SIZE8397-02、SIZE8558、SIZE8646、SIZE8824、SIZE906、SIZE9100、SIZE9364、SIZE9471、SIZE9751	XDY157、XDY160、ddy6、SIZE11309、SIZE12448、SIZE1413、SIZE4976、SIZE2020、SIZE9195、SIZE2250、SIZE2270、SIZE2365、SIZE1609、SIZE3450、SIZE3790、SIZE4161、SIZE4250、SIZE4595、SIZE4849、SIZE5929、SIZE7070、SIZE832、SIZE8558、SIZE8824、SIZE9100、SIZE9751	ddy6、SIZE12448、SIZE4976、SIZE2020、SIZE9195、SIZE2250、SIZE2270、SIZE2365、SIZE3790、SIZE4161

“/”: No MTA-based marker identified in ‘Ruixue’ × ‘Alps Otome’.

Supplementary Table 6. MTA-based SNPs for fruit weight were screened in six hybrid populations (Population A), M. domestica Borkh.'Ralls Janet' × 'Ruiyang' (RJ × RY), 'Ralls Janet' × 'YM1' (RJ × Y), 'YM1' × 'Honeycrisp' (Y × H), 'Ralls Janet' × 'Honeycrisp' (RJ × H), 'Ruixue' × 'Alps Otome' (RX × A) and *M.* prunifolia (Willd.) Borkh. 'Fuping Qiuzi'. × M. domestica Borkh.. 'Ruixue' (Q × RX). These SNPs were previously identified in two hybrid populations, M. asiatica Nakai 'Zisai Pearl' × M. domestica Borkh. 'Red Fuji'; and 'Zisai Pearl' × 'Golden Delicious'.

SNP	Population	QTL	Population	Chromosome	GDDH13 v1.1
ddy6	Q × RX	ZF_Z02.2	Z×F	Chr02	16060933
SIZE3790	Q × RX	ZF_Z02.2	Z×F	Chr02	20662023
SIZE12448	Q × RX	ZF_Z02.2	Z×F	Chr02	23876017
SIZE4161	Q × RX	ZF_Z02.4	Z×F	Chr02	27622440
SIZE8824	Y × H	ZF_F09.1	Z×F	Chr09	921113
SIZE4595	RJ × Y	ZF_Z12.2	Z×F	Chr12	15224557
SIZE4976	Q × RX	ZF_Z13.1	Z×F	Chr13	3350768
		ZG_Z13.3	Z×G	Chr13	
SIZE2020	Q × RX	ZF_Z13.2	Z×F	Chr13	4926176
		ZG_Z13.2	Z×G	Chr13	
		ZG_Z13.3	Z×G	Chr13	
SIZE9195	Q × RX	ZF_Z13.2	Z×F	Chr13	6537121
		ZG_Z13.2	Z×G	Chr13	
		ZG_Z13.3	Z×G	Chr13	
SIZE2250	RJ × RY	ZF_Z13.2	Z×F	Chr13	8952017
	Q × RX	ZG_Z13.3	Z×G	Chr13	
SIZE2270	Q × RX	ZF_Z13.8	Z×F	Chr13	11870499
		ZG_Z13.4	Z×G	Chr13	
SIZE2365	Q × RX	ZG_Z13.6	Z×G	Chr13	13933420
SIZE11309	Y × H	ZF_Z16.1	Z×F	Chr16	574182
	RJ × H				
SIZE3450	Y × H	ZF_Z16.1	Z×F	Chr16	3299638
	RJ × H	ZG_H16.1	Z×G	Chr16	

Supplementary Table 7. Significance tests of differences among genotypic groups were FDR-adjusted (False Discovery Rate). The raw P-value was derived from the significance test, and the q-value represents the FDR-adjusted P-value.

Marker-ID	populations	Test-Used	Raw P-value	q-value
SIZE2250	‘Ralls Janet’ × ‘Ruiyang’	T-test	0.037	0.0395
SIZE4595	‘Ralls Janet’ × ‘YM1’	T-test	0.025	0.0286
SIZE11309	‘YM1’ × ‘Honeycrisp’	T-test	0.047	0.0470
SIZE11309	‘Ralls Janet’ × ‘Honeycrisp’	T-test	0.012	0.0148
SIZE3450	‘YM1’ × ‘Honeycrisp’	ANOVA	0.010	0.0133
SIZE3450	‘Ralls Janet’ × ‘Honeycrisp’	ANOVA	0.000	0.0000
SIZE8824	‘YM1’ × ‘Honeycrisp’	ANOVA	0.035	0.0393
ddy6	‘Fuping Qiuzi’ × ‘Ruixue’	Mann-Whitney	0.004	0.0080
SIZE12448	‘Fuping Qiuzi’ × ‘Ruixue’	Mann-Whitney	0.008	0.0128
SIZE4976	‘Fuping Qiuzi’ × ‘Ruixue’	Mann-Whitney	0.002	0.0046
SIZE2020	‘Fuping Qiuzi’ × ‘Ruixue’	Mann-Whitney	0.001	0.0027
SIZE9195	‘Fuping Qiuzi’ × ‘Ruixue’	Mann-Whitney	0.000	0.0000
SIZE2250	‘Fuping Qiuzi’ × ‘Ruixue’	Mann-Whitney	0.000	0.0000
SIZE2270	‘Fuping Qiuzi’ × ‘Ruixue’	Mann-Whitney	0.000	0.0000
SIZE2365	‘Fuping Qiuzi’ × ‘Ruixue’	Mann-Whitney	0.000	0.0000
SIZE3790	‘Fuping Qiuzi’ × ‘Ruixue’	Mann-Whitney	0.009	0.0131
SIZE4161	‘Fuping Qiuzi’ × ‘Ruixue’	Mann-Whitney	0.008	0.0128
